# Experiential motivation and the linguistics of sitting, standing, and lying

**DOI:** 10.1002/wcs.1592

**Published:** 2022-02-02

**Authors:** John Newman

**Affiliations:** ^1^ Faculty of Arts, Department of Linguistics University of Alberta Edmonton Alberta; ^2^ Faculty of Arts, School of Languages, Literatures, Cultures & Linguistics Monash University Clayton Victoria

**Keywords:** grammaticalization, human experience, motivation, posture

## Abstract

The three human at‐rest postures of sitting, standing, and lying are basic, recurring features of human behavior and may reasonably be called primary postures. The three postures share the property of being stable through time, but they are also differentiated in terms of their overall shape, their physiological properties, and typical associated behaviors such as the association of sitting with social interaction, and lying with sleeping. The experiential realities of the three postures underlie and motivate a range of cross‐linguistic phenomena involving morphemes with meanings of “sit”, “stand,” and “lie”. The relevant linguistic phenomena include higher frequencies of occurrence compared with other kinds of posture verbs and differential behavior with respect to some morphosyntactic patterns involving notions such as agentivity. The posture morphemes can also be the source for a variety of semantic extensions reflecting experiential realities of the postures, such as the extension of “lie” to mean “sleep” in some languages. Extensions also include grammaticalizations of the posture morphemes to locative and aspectual markers which reflect the temporal stability and spatial fixedness of the postures themselves.

This article is categorized under:

Linguistics > Cognitive Linguistics
Linguistics > Language in Mind and Brain

## Graphical abstract

*Sit*, *Stand*, *and Lie* (2004): A set of three sculptures designed and created by Frances O. Cuyler (Edmonton, Canada).

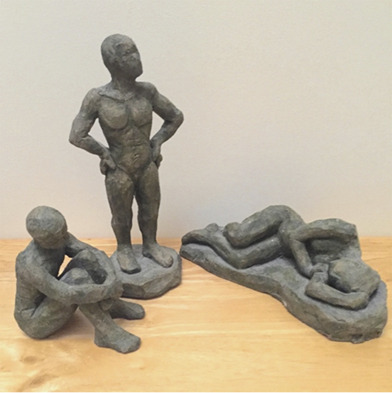

## 1 INTRODUCTION

The three at‐rest positions, or postures, of sitting, standing, and lying are part of the ordinary everyday routines of most humans, comparable to walking, eating, drinking, and so forth. The basicness of these experiences, their shared properties, and the differences between them, provide experiential grounding in varying degrees for a range of linguistic properties associated with the linguistic forms meaning “sit”, “stand”, and “lie” across languages. The linguistic facts of interest are varied and include, for example, the relative frequency in the use of the posture forms, their syntactic patterning, related lexical meanings associated with the forms, and grammaticalized extensions of these forms. In some cases, it is the experiential basicness of the postures that is a key consideration in motivating the linguistic phenomena, whereas in other cases, it is the different realities associated with each of the postures that play a key role.

Posture verbs, and “sit”, “stand”, “lie” verbs, in particular, have been recognized as a class of verbs of some linguistic interest in cross‐linguistic studies of various grammatical categories. Blansitt (1975, p. 25) had already drawn attention to the relevance of verbs with meanings of “sit”, “stand”, and “lie” in patterns of progressive aspect marking in the world's languages. Aikhenvald (2000), a typology of noun categorization devices, references posture verbs, particularly “sit”, “stand,” and “lie”, at various points throughout her book. Similarly, in Aikhenvald and Dixon's (2007) volume of chapters on the typology of serial verb constructions, the same posture verbs are identified as a relevant category of verbs, particularly in the minor verb slot of serial verb constructions. Posture verbs have also played a special role in the cross‐linguistic typology of locative expressions in the framework of Ameka and Levinson (2007), as discussed further in Section 4.3.

In the cognitive linguistic approach to posture verbs adopted in this overview, the particular focus will be on exploring motivations for various patterns involving the posture verbs. This includes the goal of finding motivations for semantic shifts and grammaticalizations, as well as motivations for patterns involving the posture verbs used in their literal postural meanings. Section 2 briefly reviews the place of motivation in the recent history of linguistics; Section 3 summarizes the main experiential realities relating to these postures; in Section 4, I present a selection of linguistic facts and their experiential motivations; Section 5 is the conclusion.

## 2 MOTIVATION IN LINGUISTICS

Finding motivations for linguistic phenomena has always been a part of modern linguistics, reflecting our desire to seek explanations for human behavior generally. Motivations that have been proposed can be categorized, somewhat simplistically, as “internal”, that is, deriving from considerations of a language as a system of units and their relationships to one another, or “external”, that is, based upon connections between language and other realms of human behavior such as communicative needs, social settings, cultural practices, and psychological processes. Specific examples of the former would be symmetry within the vowel system to explain vowel inventories, analogy as a motivation for leveling in a morphological paradigm, and system pressure to motivate vowel shifts such as push‐chains and pull‐chains (Aitchison, 2001, pp. 153–168). Examples of external motivations would be the communicative principle that more surprising or disruptive information precedes more continuous or predictable information (Givon, 1984, p. 206), the reliance on properties of the human vocal tract, and human perception as motivation for phonological patterns and sound change in the Natural Phonology framework (Donegan & Nathan, 2014; Donegan & Stampe, 2009), and sociolinguistic causes of change (Aitchison, 2001, pp. 133–152). Extralinguistic motivation is common, too, in accounts of the interrelatedness of senses in cases of word polysemy: “The polysemy of words connects most of them with many different items of experience, and many different spheres of experience,…” (Stern, 1931, p. 119).

In the last quarter of the 20th century, a “cognitive turn” in linguistics can be seen which sought to identify the cognitive underpinnings of a wide range of linguistic phenomena, including phonological patterns, syntactic patterns, lexical semantics (especially polysemy), and discourse phenomena. This new approach to finding external motivation of linguistic phenomena came to be called “cognitive linguistics” and found a home in the International Cognitive Linguistics Association (ICLA), founded in 1989, along with the journal *Cognitive Linguistics*, established at the same time. Important influential works of this movement included Langacker (1987, 1991), Lakoff (1987), and Talmy (2003a, 2003b). In these works, identifying motivations for linguistic phenomena from within the broad cognitive faculty was a central concern. An overview of the types of motivation that cognitive linguists have concerned themselves with can be found in Radden and Panther (2004). Not all linguists are sympathetic to the cognitive linguistic approach. In particular, linguists who follow more formalist theories, such as generative linguistics, have tended to avoid questions of extralinguistic motivation and tend to be skeptical about the explanatory value of “external motivations” (cf. Newmeyer, 2000; Radden & Panther, 2004, p. 14). One should note, though, Ruwet's (1991) attempt to introduce human experience as an explanatory factor in syntactic theorizing as practised by generativists in the 1970s and 1980s.

The kind of motivation that is explored below amounts to identifying experiential realities of the key human at‐rest positions and considering these realities as factors that have shaped the linguistic patterns involving the morphemes with the meanings of “sit”, “stand”, and “lie”. Some of the linguistic patterns involve completed historical shifts in meaning and use. In such cases, there are varying degrees to which modern‐day speakers are aware of the original postural sense and/or are aware of the experiential realities originally motivating the historical change. The experiential realities do not predict the linguistic facts in any absolute sense, but can be put into correspondence with the linguistic facts and provide in varying degrees some rationale for the linguistic facts.

## 3 SITTING, STANDING, AND LYING

The experiential realities that are of interest in the present context are the everyday experiences of being at rest: sitting, standing, and lying. These three postures may be called the primary postures. These experiences are to be understood as states that are maintained, in contrast to the actions of moving into or out of these states. Unless we are performing energetic physical exercises, we are not in the habit of being in these states for merely an instant, but rather we are accustomed to maintaining these postures for longer, ranging from minutes to hours at a time. There are many variations on exactly how one can be in these positions (sitting on a chair vs. sitting on the floor; lying on a bed vs. lying on the floor, sitting cross‐legged vs. sitting with legs outstretched, etc.) and clearly, there are different cultural preferences for particular postures. For the present purposes, however, it is sufficient to characterize the three postures in the simplest terms: sitting is a posture in which the upper torso is held vertical while the buttocks rest on and are supported by a horizontal surface (or the buttocks are close to a horizontal surface in some forms of squatting); standing is a posture in which the whole torso is upright and the soles of the feet (or covering of the soles) are in contact with, and are supported by a horizontal surface; lying is the posture in which the body rests on, and is supported by, a horizontal surface (such as bed or ground) with the body extended horizontally along this surface (Box 1).

BOX 1 **An anthropological view of human posture**

A classic anthropological study of human posture is Hewes (1955), who reviewed about 100 human postures from 480 different cultures, mainly focused on sitting, kneeling, crouching, and squatting positions, plus some standing positions (lying postures were excluded from the study due to insufficient amount of information). Hewes' study revealed the great diversity of postural habits, the religious significance of postures, gender differences between preferred postures, relationships between types of labor and posture, and so forth. Hewes documented the major postural types to be found across cultures: one‐legged resting stance; chair‐sitting postures; deep‐squatting postures; sitting with legs stretched out; cross‐legged postures; kneeling on knees; sitting with legs folded to the side; one knee up, the other leg down and flexed. Standing with one or both hands on the hips was reported to be a common informal pose for both sexes, and across many cultures. Similarly, folding the arms across the chest while standing was reported to be very common. Hewes (p. 242) also added an interesting comment on the lexical resources in the world's languages for describing postures: “The English postural vocabulary is mediocre—a fact which in itself inhibits our thinking about posture. Quite the opposite is true of the languages of India, where the yoga system has developed an elaborate postural terminology and rationale, perhaps the world's richest.”

One can identify obvious realities associated with these postures without the need for expert opinion (Newman, 2002a, pp. 1–3; Kinn et al., 2018). The main properties we can identify are summarized in (1).

(1) a. The vertical extension reached is the highest with standing and the lowest with lying.

b. Standing is higher than wide, lying is wider than high, and sitting is relatively compact.

c. Most balance and sensorimotor control is required for standing, less so with sitting, and no such balance or control is required with lying.

d. All three postures can be maintained in comfortable ways, varying from minutes (standing), to hours (sitting, lying).

More scientifically based observations can also be made about the postures from experimental work. In an experimental study of human energy expenditure associated with the postures of sitting on a chair, standing on the ground, and lying on a bed, Amaro‐Gahete et al. (2019) confirmed that heart rate is significantly higher for standing than for sitting and significantly higher for sitting than for lying. However, when a number of other measures are considered together (ventilation, respiratory rate, etc.), it was found that standing was associated with significantly higher expenditure than the other two postures, but there was no significant difference in energy expenditure between sitting and lying postures. That is, energy expenditure overall leads us to distinguish standing from the other postures. Biddle et al. (2015, chap. 16), a chapter on the psychology of sitting, similarly makes a separation between standing (and moving) on the one hand and sitting and lying on the other, in their review of energy expenditure associated with postures and motion. Gibbs et al. (1994) established key cognitive notions (image schemas) associated with the standing posture through an experimental study that involved, among other things, having participants engage in various bodily exercises facilitating their conscious reflection on standing. Balance, verticality, center‐periphery, resistance, and linkage were the five main image schemas that emerged in participants' responses to specific questions on each of these schemas. The postures are also associated with various social, communicative, and behavioral functions which will differ in their detail from one culture to another. It is sufficient for the present purposes to observe that all three postures permit verbal communication and free‐hand movement while maintaining the posture.

While accepting that there are cultural variations as to precisely how one typically sits, stands, and lies, the three postures, characterized in the rather schematic way we have done here, can be viewed as a relatively basic feature of human experience, in the same way that certain activities (walking, grasping, talking, etc.) may be viewed as basic. The medical literature cited above (Amaro‐Gahete et al., 2019) also appeals to just these three postures in their study of at‐rest positions, without the authors seeing any need to justify why these three and only these three positions are the focus of the study. The three human postures represent, arguably, “conceptual archetypes” (Langacker, 1991, p. 294): “[cognitive semantics] recognizes that certain recurrent and sharply differentiated aspects of our experience emerge as prototypes, which we normally use to structure our conceptions insofar as possible.” Langacker's (1991) further comments on prototypical structures in grammar and extensions from prototypes (see Goldberg, 2014, for more discussion of event archetypes and their reflexes in grammar):

Since language is the means by which we describe our experience, it is natural that such archetypes should be seized upon as the prototypical values of basic linguistic structures. Extensions from the prototype occur for the same reasons that they do with lexical items because of our proclivity for interpreting new or less familiar with reference to what is already well established… (p. 295).

Whether the three postures commonly characterized as sitting, standing, and lying constitute conceptual archetypes in every community or social group remains an open question. Some at‐rest human positions will be a basic feature of everyday life for all humans, though clearly there are cultural variations on preferred postures, as discussed in Hewes (1955). The focus in this overview on verbal predicates relating to sitting, standing, and lying, sometimes referred to as the “cardinal posture verbs”, reflects the focus found in the literature under review.

## 4 LINGUISTIC PROPERTIES OF “SIT”, “STAND”, “LIE” MORPHEMES

### 4.1 Form and frequency of literal “sit”, “stand”, and “lie”

There is a close association between the action of moving into a posture and the resulting at‐rest state: we typically move into the posture positions in order to maintain those positions for some time, though this is admittedly more obvious with lying and sitting. The close relationship between entering the posture state and maintaining the state is reflected in the fact that languages may derive the state expression from the action expression or vice versa. In the Australian language Ngan'gityemerri (Daly family of the Northern Territory, Australia), the stative postural verbs are basic and the action counterparts are formed by means of a “go” + posture verb construction (Reid, 2002, pp. 243–244). Alternatively, the action verb may be the more basic one, as in Usan (Trans–New Guinea, Papua New Guinea), where the action verbs *bugâb* “sit down”, *naget* “stand up”, and *inâb* “lie down” are basic and the at‐rest meanings are expressed by putting the action verbs into a continuative aspect (Reesink, 1987, 132). In English, the three primary at‐rest meanings are most closely associated with the verbs *sit*, *stand*, and *lie* (without accompanying verb particles *up* or *down*), while the corresponding dynamic change‐of‐state senses are most closely associated with the same verbs used with verb particles, that is, *sit down*, *stand up*, and *lie down*. The semantic distinction between the forms with and without verb particles is, however, not entirely clear‐cut in usage. So, for example, the imperative forms *Sit!* and *Stand!*, without the particles, can be used as (rather brusque and official‐sounding) commands, as might be used, for example, by the principal at a school assembly addressing pupils to mean “sit down” and “stand up”. The forms with particles are also commonly used with the implication that the resulting at‐rest state is maintained, as in *We need to sit down and have a chat* [cf. the discussion of implied maintenance of position with Lao *nang* “sit” in Enfield (2002, pp. 35–37), and Japanese and English “sit” constructions in Newman and Yamaguchi (2002)]. Newman (2009, pp. 41–44) reviews the factors favoring one or the other of the static versus dynamic interpretations of English *sit*. Talmy (2003a, pp. 78–86) reviews lexicalization patterns of postural morphemes and considers patterns of lexicalization involving not just stative meanings (e.g., “to be sitting”) and inchoative meanings (e.g., “to enter into a state of sitting”), but also the causative or “agentive” meanings (e.g., “to cause someone to be seated”). The causative plays a role, for example, in the construction of the German inchoative verb *sich setzen* “to sit oneself down”, a reflexive form of the causative *setzen* “to set something/someone down”, which in turn is historically derived from the stative *sitzen* “to sit” (parallel facts for the German “lie” verb).

Noonan and Grunow‐Hårsta (2002, pp. 81–82) discuss the three primary postural meanings in Chantyal (Tibeto‐Burman, Nepal) and find experiential motivation for different formal properties of the posture expressions. The relevant facts they report are: “stand” can be expressed as a simplex verb (i.e., not further reducible into smaller morphemes), either *yep‐* “maintain a standing position” or *yes‐* “assume a standing position”; “sit” is also expressed as a simplex verb, *ci*; “lie”, on the other hand, can only be expressed as a complex form consisting of multiple words, for example, *terso la‐* “maintain a horizontal position”, *terso ta‐* “assume a horizontal position”, and so forth (the authors list seven options). The authors attach some significance to this set of facts, relating to differential degrees of agentivity among the postures:

This distribution of simple and complex expressions is consistent with predictions from Givon's (1984) time‐stability continuum and Hopper and Thompson's (1980) transitivity continuum: “lie”, the least agentive and therefore least transitive of the basic posture expression is the least prototypically verb‐like and therefore the one most likely to receive complex expression involving a stative positional word. (p. 82)

The related language Magar (also Nepal), also discussed by the authors, shows a similar lexical pattern whereby “stand” and “sit” may be expressed as simplex verbs, but there is no simplex or even complex (!) verb simply meaning at‐rest “lie” (Noonan & Grunow‐Hårsta, 2002, pp. 90–92).

There may be special properties associated with the forms of three postures, or the three posture verbs along with some other basic verbs, reflecting the unity of the postures. In the Australian language, Ngan'gityemerri referred to above, for example, there are just six “simple” intransitive verbs that share the property that they may be used as complete verbal units without being accompanied by a co‐verb (Reid, 2002, pp. 241–252). The six verbs include all three cardinal posture verbs and three others with meanings “perch”, “go”, and “travel”. Foreman and Lillehaugen (2017, pp. 271–277) identify a class of “positional” verbs in Colonial Valley Zapotec, a historical variety of Zapotecan languages (Mexico) with special formal properties. Their class of positional verbs includes the cardinal posture verbs “sit”, “stand”, and (less frequently) “lie”, together with a few other verbs glossed as “be contained”, “be sticking”, “be floating”, “be positioned across”, and so forth. Only this class of positional verbs can appear with “zero‐marked stative” morphology (pp. 277–285). In English, *sit*, *stand*, and *lie* verbs are distinguished from other at‐rest verbs by their higher frequencies of occurrence in English corpora (Newman & Rice, 2001). Newman (2009) reports on the frequencies of selected posture verbs based on the SemCor corpus, a corpus of English that has been manually tagged using WordNet 3.0 word senses (Fellbaum, 1998; Landes et al., 1998). In this system of semantic tagging, stative senses of the three posture verbs are distinguished from their dynamic senses. Table 1 summarizes the findings, reporting frequencies of each posture verb as a lemma, that is, all inflected forms of each verb (the lemma for *sit*, for example, includes *sit*, *sits*, *stood*, and *standing*). As can be seen in the table, *sit*, *stand*, and *lie* are by far the most frequent of the posture verbs, a reflection, arguably, of the more salient role these three postures play in our everyday experience. Some caveats apply, however, the meanings for some verbs (other than *sit*, *stand*, *lie*) shown in Table 1 cover both at‐rest and the dynamic senses (inflating the frequency); human versus nonhuman subjects are not always distinguished; the results report on language use in a sitting (as opposed to squatting) culture.

**TABLE 1:** Frequencies of English posture verbs in the semantically tagged SemCor corpus, based on Newman (2009, p. 35)

Verb lemma	Wordnet sense	Frequency
*stand*	be standing, be upright	133
*sit*	be sitting	124
*lie*	be lying, be prostrate; be in a horizontal position	46
*hang*	be suspended or hanging	27
*lean*	incline or bend from a vertical position	19
*squat*	sit on one's heels	8
*kneel*	rest one's weight on one's knees	7
*crouch*	sit on one's heels	4
*stoop*	bend one's back forward from the waist on down	4
*sprawl*	sit or lie, with one's limbs spread out	4
*perch*	sit, as on a branch	4
*bend*	bend one's back forward from the waist on down	3
*lounge*	sit or recline comfortably	2

*Note*: The frequency counts include all the inflected forms of each verb.

### 4.2 Syntax and morphosyntax of literal “sit”, “stand”, and “lie”

A number of studies investigate specific properties of language structures in which the cardinal posture verbs occur in close proximity to, and in construction with, other verbs, and report on the richness and complexity of such combinations. The ease with which the posture verbs enter into constructions with other verbs reflects the experiential reality that maintaining the postures allows for simultaneous actions (communication, handling of objects) with sitting being the customary position for much social interaction. Aikhenvald and Dixon (2007) identified the role of posture verbs as the minor verb in serial verb constructions where actions are performed while maintaining a certain posture. Enfield, 2002, pp. 34–38) discusses properties of what he calls an “associated posture construction” in Lao, that is, a construction consisting of posture verb and action verb, and how the meaning of this construction differs from simple action sequences like “fall” + “break”. In their study of “posture verb pseudo‐coordination”, based on corpora from Danish, Norwegian, and Swedish, Kinn et al. (2018) found different associations for each posture verb: “sit” is most associated with written and spoken communication, consumption, and manual activities performed with the hands; “stand” is most associated with foot motion, maintaining or regaining balance, vision, and communication; “lie” is most associated with sleep, rest, inactivity, helplessness, deterioration, and death. The authors argue for the idea that the posture itself “facilitates” concomitant events, that is, the posture provides “the required stability of posture and Location for a certain Duration” (Kinn et al., 2018, para. 100). Similar findings are reported in Newman and Rice's (2004, pp. 365–372) study of an English “simultaneous conjunction construction” *sitting/standing/lying and* V*‐ing*, based on the British National Corpus. The constructions that the posture verbs enter into can be specific to just the three posture verbs, reflecting the uniqueness and unity of the primary postures. Early (2000, pp. 87–88), for example, draws attention to a preference for using the three primary posture verbs of Lewo (Oceanic, Vanuatu) *to* “sit”, *su* “stand”, *mo(no)* “lie, stay, dwell” in some constructions, especially serial verb constructions. He notes, for example, the use of precisely these three verbs in a “wait for someone/something” construction. In the absence of any independent lexical word “wait”, each of the thee posture verbs combines with a bound morpheme *‐mate* “wait” to express the “wait” meaning: *to‐mate* “sit‐wait”, *su‐mate* “stand‐wait”, and *mo‐mate* “lie‐wait”. In Yuma (Yuman family, Arizona and California), also known as Quechan, the three primary posture senses play a key role in an associated posture construction. In this construction, posture can be indicated in medio‐passive verb forms by prefixes *t‐* “to do while sitting”, *v‐* “to do while standing”, and *a‐* “to do while walking or lying” (Halpern, 1946, p. 274).

Some morphosyntactic facts in languages would appear to correlate with the gradation in degree of control associated with the three postures (Rice, 2002, pp. 62–66; Newman, 2004, pp. 209–213). In Manam (Oceanic, Papua New Guinea), there is an interesting way in which *tui* “stand” is distinguished from *soaʔi* “sit” and *eno* “lie/sleep” (Lichtenberk, 1983, pp. 219–220). Manam distinguishes “active” and “stative” verbs. Active verbs are described by Lichtenberk as having agentive subjects and include verbs with meanings “go”, “work”, “jump”, “speak”, “hit”, “give” and *tui* “stand”. A characteristic of Manam active verbs is that the reduplication of these verbs is associated with progressive aspect, meaning that the event was in progress at the time of speaking or at the time of another event. Stative verbs, on the other hand, are described by Lichtenberk as having patient subjects and have meanings related to states or changes of state, with meanings such as “be big, grow big”, “be bad, become bad”, “be broken, break (intr.)”, as well as *soaʔi* “sit” and *eno* “lie”. When reduplicated, the state verbs are associated with habitual, not progressive, aspectual meaning. In Manam, then, the “stand” verb is aligned with verbs of action involving agents, whereas the “sit” and “lie” verbs are aligned with verbs describing states without agents.

### 4.3 Locative extensions

The human posture verbs may be extended to refer to the location of other animates or inanimates, as in English *Our house stands on the corner*. A variation on this development is an extension to existential constructions, which can be considered a nonspecific kind of location. In Mbay (Nilo‐Saharan, Chad), for example, a locative/existential expression equivalent to “here is/are, there is/are X” builds upon the three posture verbs by requiring one of the adverbs: *ndìn* for an object viewed as sitting (from *ndì* “sit”), *ɗàn* for an object viewed as standing (from *ɗà* “stand”), and *tèn* for an object viewed as lying (from *tò* “lie”; Keegan, 2002, p. 349). These extensions are presumably motivated by the model of fixedness in one location that the primary human postures present. Often, however, the extension to a general locative meaning involves some qualification in terms of the types of entities that can be located, related to some experiential realities of the human postures. In other words, the use of the posture morphemes in locative/existential constructions may not completely displace the original posture meaning and it can be difficult to know from dictionaries and grammars exactly what is, and what is not, accepted by speakers concerning these extended uses. Accounts by linguists who report their own native‐speaker intuitions about such extensions are therefore particularly valuable in this regard (cf. Atintono, 2012; Lemmens, 2002; Song, 2002), as are accounts by linguists reporting on native speakers' responses to targeted questions about such extensions (cf. Rice, 2002).

In some cases, the locative extension is influenced by the resemblance to the overall shape of a human posture, for example, the verticality and height‐to‐width ratio of a tree motivate the use of “stand” to describe the location of a tree, just as the horizontality and dimensions of a snake make motivate the use of “lie” to describe a snake's location. It is clear, however, that the full range of locative/existential extensions allowed with each of the primary postures cannot be explained away simply by comparison with the overall shapes of the human postures (see Serra Borneto, 1996, for extensive discussion of the perceptual and cognitive factors that play a part in these choices). To take a couple of examples, Reid (2002, pp. 246–247) discusses motivations for extensions of “stand” in Ngan'gityemerri to objects above the ground that are supported by legs or leg‐like supports, for example, centipedes and cars, while things not raised above the ground, such as roads and felled trees, use the “lie” verb. Lizards and crocodiles, however, are used with “lie” even though they have legs and can raise themselves above the ground. In the case of these animals, Reid argues, they are conceptualized as being prototypically in contact with the ground and hence “lie” is the appropriate verb. In other words, habitual or prototypical behavior of the animal or insect, not just overall shape, can play a part in the extension. Similarly, Kaufman (2013) discusses the role of the speaker's perspective in Biloxi (Ohio Valley Siouan, USA, now extinct), where “stand” is used in reference to a single house, but “lie” when referring to multiple houses, as in a village or town. Kaufman (p. 290) explains: “Although a single house has a vertically salient axis to a viewer from close range, several houses scattered about may be considered a single group that can be scanned and perceived horizontally when viewed from a distance.” Serra Borneto, 1996, pp. 468–470) makes a similar point in his discussion of the choice of German *liegen* “lie” with reference to a town or a mountains, where the town or mountain is conceived of as a spot on a map (a “geotopographical location”) rather than a vertical entity with a base.

There can be other factors that influence extensions to locative/existential constructions, apart from shape and (prototypical) orientation. Lemmens (2002, pp. 108–117) argues for the idea of “containment” or “contact” as relevant to the locative extensions of Dutch *zitten* “sit”. Lemmens reports that almost 50% of the occurrences of *zitten* in the corpus he used could be interpreted as real or metaphorical containment, locating one entity within the confines of another. Lemmens (p. 109) suggests that the idea of close contact and containment associated with sitting in a confined space such as a chair has been extended via a kind of metonymy to the broader concept of a larger enclosing space. In Manam, *soaʔi* “sit” and *eno* “lie” (but not *tui* “stand”) can both be extended to constructions that can be understood as locative (when the subject is definite) or existential (when the subject is indefinite). Lichtenberk (1983, p. 496) describes the distribution of the two verbs in this usage as follows: “*soaʔi* is used with subjects whose referents are higher animals and boats at sea; *eno* is used elsewhere, including boats viewed close up.” In Lewo, *su* “stand” is associated with general existence, *to* “sit” temporary or short duration, and *mono* “lie” extended or permanent location (Early, 2000, p. 90).

Finally, one should note the special role of posture verbs, especially the cardinal posture verbs, in the typology of locative constructions as described in Ameka and Levinson (2007). In their methodology, speakers were asked to answer the question “Where is X?” in a relatively colloquial manner and the preferred structure of the responses from speakers of a language constitutes the Basic Locative Construction (BLC) of that language. All the contributions to the 2007 Special Issue of the journal *Linguistics* introduced by Ameka and Levinson use the same, or partially similar, visual stimuli materials of pictures in order to identify a BLC. Ameka and Levinson (p. 852) established four language types using this method, summarized as:

- Type 0: no verb in the BLC.
- Type I: single locative verb in the BLC.
- Type II: large or unlimited set of positional verbs in the BLC.
- Type III: small, contrastive set of (human) posture or positional verbs in the BLC.

Ameka and Levinson elaborate further on these types, noting that Type I can include one or two verbs in a BLC, Type II can include six or more (up to possibly 200) verbs, and Type III includes three to six verbs. For Type III, the authors single out the three key at‐rest postures (lending further support to the idea that these three postures are conceptual archetypes): “These verbs typically draw on the human posture verbs “sit”, “stand”, “lie”, but often also incorporate less anthropomorphic positionals like ‘hang’” . Among the articles in the Special Issue, Guirardello‐Damian (2007) reports on Trumai (language isolate, Brazil), a Type III language; Hellwig (2007) reports on Goemai (Western Papuan outlier, North Moluccas of Indonesia), identified as mainly a Type III language, but with some Type II characteristics; Kutscher and Schultze‐Berndt (2007) report on German, identifying it as a Type II language, albeit one making extensive use of “stand” and “lie” verbs (cf. Serra Borneto, 1996).

### 4.4 Aspectual extensions

The idea of some temporal extension is inherent in the maintenance of a human posture. The inherent temporal extension of the primary posture verbs motivates extensions to markers of a progressive or other progressive‐like aspect. The overview of aspectual extensions in Newman, (2002b, pp. 16–17) reveals quite a range of differently labeled aspectual categories relevant to the extensions of the posture verbs, including progressive, continuous, habitual, durative, iterative, and persistive aspects. There is a tradition of distinguishing “progressive” and “continuous” aspectual categories, with the former reserved for dynamic verbs involving an agent and the latter reserved for when there is no volitional agent involved, but the demarcation between the two terms is not always a very clear one (Bybee et al., 1994, pp. 133–137; Mair, 2012). Kuteva (1999) provides numerous examples of aspectual extensions; there are also detailed discussions of such extensions for specific language families, in particular Siouan (Rankin, 2004; Watkins, 1976), and Oceanic (Early, 2000; Lichtenberk, 2002). Extensive discussion of aspectual (progressive, durative, or habitual) extensions of the Dutch posture verbs *zitten* “sit”, *staan* “stand”, and *liggen* “lie” can be found in Lemmens (2002, 2005, 2017). In a study comparing alternative progressive constructions in Dutch, Lemmens (2017, pp. 178–182) found that the progressive aspect arising from the extension of the posture verbs favored stative, atelic main verbs, while the progressive aspect built on the prepositional construction with *aan* “at” favors telic main verbs. Lemmens (2005, pp. 196–209) provides an insightful analysis of the (corpus‐based) combinatorial preferences of each of these posture verbs functioning as aspectual auxiliaries and their associated main verbs in Dutch. *Wachten* “wait” was found to be the most frequent main verb occurring with each of the three postural auxiliaries (Lemmens, 2005, p. 197), consistent with the overall preference for stative, atelic verbs mentioned above and reminiscent also of the close bond between the posture verbs and the morpheme “wait” in Lewo, discussed in Section 4.2. *Staan* “stand” was found to occur with the widest range of main verbs (especially, movement verbs), while *zitten* “sit” has progressed furthest in terms of semantic bleaching to pure aspectual meaning. Hilpert & Koops (2008, pp. 243–246) review the (synchronic and diachronic) properties of the pseudo‐coordination construction *sitta* “sit” + verb in Swedish, where the semantic contribution of *sitta* can be mainly aspectual (progressive or durative), or, under certain conditions, mainly postural.

In addition to direct motivation for aspectual extensions from the circumstances of the postures themselves, the locative extension of the posture verbs discussed in the previous extension may itself be a possible intermediate stage in an extension to aspectual marking, closely tied to the idea that the thing being located in space continues to be in that location over some time. Kaufman (2013, p. 289), for example, comments on the co‐presence of locative and aspectual functions in a discussion of examples in Biloxi, referred to above, where the posture verbs are being used with inanimate objects: “… the actual physical configuration of the item also implies that it continues to remain in that position, and the verb is therefore also serving as an aspectual.” Kuteva (1999, 2001) argues convincingly for an extension of the posture verbs to a general locative use as being a transitional stage in the evolution of the aspectual marking of these verbs. Still, she acknowledges that the aspectual extension may take place even without such an intermediate stage, as appears to be the case with the progressive aspectual extension of Korean “sit” and “lie” (Song, 2002, pp. 378–379; Kuteva, 2001, p. 73). Jarad also presents evidence for the progressive aspectual extension of a participial verb based on “sit” functioning as a progressive marker in Emirati Arabic, without any intermediate stage of “sit” functioning as a locative marker (Jarad, 2015, p. 93). Rankin (2004, p. 203 and p. 225), too, views the continuative aspectual extension of the posture verbs in Siouan as evolving directly from the posture senses.

As with the locative extensions of these verbs, the choice of posture verb as an aspectual marker can be influenced by various experiential realities associated with the postures. In Mbay, already referred to above in connection with locative/existential extensions, the cardinal posture verbs can be used as auxiliaries marking progressive aspect, but the original meanings of the posture verbs can still be present. In (2a), the narrator presumably understands the child as being seated and *ndì* “sit” contributes this meaning along with signaling the progressive aspect. In other cases, *ndì* “sit” may be used as the preferred choice of progressive auxiliary where the main verb refers to action or movement (2b) or where the subject is nonhuman (2c).

(2) a. Ngōn ndì tōń  ngōn‐jī‐ǹ.

child sit licking small‐hand‐his

“The child is licking his finger (seated).” (Keegan, 2002, p. 346)

b. Ndì āw ɗá? Ndə̀ m‐āw súkū‐u.

sit you.go where? sit I‐go market‐LOCATIVE

‘Where are you going? I am going to the market.’ (Keegan, 2002, p. 347)

c. Dɔ̀‐ḿ ndì ndàa.

head‐my sit becoming.white

“My hair is turning white.” (Keegan, 2002, p. 348)

Langdon (1978) reviews data on auxiliary formation in Yuman languages (Baja California, California, Arizona). A small set of just nine verbs are used as auxiliaries throughout the Yuman family to characterize an imperfective meaning, that is, ongoing event or action in progress. When used as the main verb in a verb + auxiliary construction, the verbs have the meanings “sit”, “stand”, “lie”, “stay‐PL”, “be there‐PL”, “arrive”, “go”, “come”, and “make noise”, with “sit” being the most general in usage and labeled by Langdon as the “neutral” auxiliary. Langdon refers to all nine verbs used as auxiliaries in this way as “locational” auxiliaries, argued to be present as both full verbs and as auxiliaries in reconstructed Proto‐Yuman. As with Mbay, the choice of one of these auxiliaries over another is influenced to some degree by its compatibility with the primary verb. Langdon explains the choice of auxiliary as follows:

…primary verbs meaning “sit”, “stand”, and “lie” would have obligatorily required the locational auxiliary with the same basic meaning, but verbs meaning “eat”, “know”, or any other activity not dependent on a particular position or motion could select more freely on the speaker's choice depending on what element of the situation he wished to select for overt expression. (Langdon, 1978, p. 105).

### 4.5 Article/demonstrative extensions

The posture verbs may be historical sources for demonstratives and definite articles (Heine et al., 2020, pp. 416–419). In Hoocąk (Mississippi Valley Siouan, USA), also known as Winnebago, the three posture verbs *nąk* “sit”, *jee* “stand”, and *ąk* “lie” combine with a proximal (*‐re)* or a distal (‐*ga*) deictic morpheme to form a new paradigm of demonstratives (Helmbrecht, 2017, pp. 150–151), as in *nąka* (from *nąk‐ga*) “that (sitting/neutral position; distal)”, *jeega* (from *jee‐ga*) “that (standing/vertical position; distal)”, and *ąka* (from *ąk‐ga)* “that (lying/horizontal position; distal)”. These demonstratives incorporate shape/configurational differences, rather than proximate versus distal differences. A proximate versus distal function of posture verbs as demonstratives is, however, reported for some Australian languages by Evans (1990):

Demonstratives of stance‐verb origin will be drawn from the “stand”, “sit”, “lie” set in such a way that the imputed distance is represented iconically by the stance verb most appropriate to the height of the object at various distances. The exact value will depend on the number of stance verbs feeding the system, but demonstratives deriving from “lie” will typically be distal or invisible, and demonstratives from “stand” will be proximal. “Sit” is the most common origin, and in systems where it provides the only stance‐verb‐derived demonstrative its interpretation may be distal, intermediate, or proximal. (p. 142)

The cardinal posture verbs of Euchee (language isolate of Oklahoma, USA), *ci* “sit”, *fa* “stand”, and *’e* “lie”, form the basis for a three‐way noun classification system, as discussed in Linn, 2000, pp. 364–370). Each of these forms may be attached as a clitic to an inanimate noun at the end of a noun phrase indicating definiteness or location, translated usually as “the”, “this”, or “that”. Examples are given in Table 2. In some cases, the shape of the referent has some clear resemblance to one of the primary postures: vertical elongation of an object such as a tree or a door is associated with “stand”, horizontal elongation such as a log is associated with “lie”, roundish things such as bullet and rock are associated with “sit”. In other cases, however, there is no obvious physical resemblance, for example, *dzetapaci* “my strength” takes the “sit” clitic, *‘agafa* “the day” takes the “stand” clitic, and *yagokwene'e* “the song” takes the “lie” clitic. The “sit” class is the default class and is used for things of no definite shape (Linn, 2000, p. 366). For the most part, an inanimate noun is assigned to one and only one of the three classes, reflecting an “inherent” position of the object, but there are some cases where alternatives are possible. For example, *ya* “tree” (also transcribed by Linn sometimes as *‘ya)* occurs with the “stand” clitic when referring to a tree that is standing and alive, but it occurs with the “lie” clitic when referring to a fallen tree, that is, a log. Similarly, *dowõne* (where *do‐* is a first person singular prefix) can occur with the “sit” clitic in which case it means “my spirit”, whereas when used with the “stand” clitic, it translates as “my shadow”. Also, Linn reports that there is room for some playfulness in how nouns are assigned to a class, citing an example where the word for “nose” was humorously assigned to the “stand” class when it would normally be assigned to the “sit” class. Kaufman (2013) and Helmbrecht (2017) discuss similar phenomena involving the use of “sit”, “stand”, and “lie” as modifiers of nouns, especially as classificatory demonstratives, in Siouan languages. Rankin (2004, p. 225) views the extension of the posture verbs to locative or aspectual markers in Siouan languages as a necessary intermediate stage in the evolution of the article/classifier use of these verbs (the cardinal posture verbs in Euchee, a language isolate, also function as locative markers).

**TABLE 2:** Examples of the three noun classes of Euchee, based on Linn (2000)

ci “sit” class	fa “stand” class	’e “lie” class
thlaci “the bullet”	yafa “the tree, alive”	ya'e “the fallen tree”
tici “the rock”	yadash'ifa “the door”	sha'e “the field”
dowõneci “my spirit”	chyakafa “the can”	dowõne'e “my shadow”
k'ondici “the meat”	kafifa “coffee in the mug”	John'e “(the name) John”
dzetapaci “my strength”	’agafa “the day”	yagokwene'e “the song”

*Note*: *k’*, *sh’*, *‘y* represent glottalized consonants, elsewhere*’* represents a glottal stop. *õ* is a nasalized vowel.

### 4.6 Miscellaneous extensions

The posture verbs are often associated with other polysemous senses that can be understood as motivated by the experiential realities. Newman (2002a) and the individual chapters in Newman (2002b) contain examples of this kind of polysemy in various languages. *“*Stand” morphemes for example, are often extended to uses where strength, resilience, resistance, support is involved, as in *stand by someone*, *stand eager to help*, *stand for the truth*, *I cannot stand loud noise*, etc. This kind of extension corresponds to the physical reality of standing as the posture associated with most energy expenditure and sensorimotor control. “Sit” morphemes maybe extended to “dwell”, “live” senses, reflecting the durative nature of postures and possibly a reflection of sitting as a kind of default posture. “Lie” morphemes may be extended to “sleep”, “camp”, “have sexual intercourse with”, and so forth, reflecting common experiential associations. Another grammaticalization that should be mentioned is the extension to a copula use, as in the development of Spanish *estare* “be” from Latin *stare* “stand” (Batllori & Roca, 2012). Lesuisse and Lemmens (2018, pp. 61–65) review the use of the three posture verbs as copulas in Early and Late Modern English and discuss the semantic dimensions of the predicative adjective in such constructions. They found that the adjectives used with *sit* are associated with intellectual activity, attainment, and inactivity, weakness; in the case of *stand* with power, dominance, resistance; and in the case of *lie* with lethal harm, death, weakness, and inactivity. Breeuwer's (2019) diachronic study of *sit*, *stand*, and *lie* investigated the relationship between the decrease in the use of English posture verbs (without differentiating locative and copula uses) in the Modern English Period and the concomitant increase in the use of the *be* + V‐*ing* in the same period.

The contrast between standing and lying postures in their physical attributes and associated behavioral patterns is particularly striking (cf. Lemmens, 2002, pp. 117–122). The contrast is reflected in the now extinct Chitimacha (language isolate of Louisiana, USA), where the two posture auxiliaries *či(h)* “standing” and *pe(h)* “lying” function as honorific morphemes. When applied to humans, the “standing” morpheme is associated with respect and the “lying” morpheme carries derogatory or abusive nuances (Swadesh, 1946, p. 322). The same postural differences motivate the difference in the locative/existential use of *soaʔi* “sit” used with higher animate subjects such as pigs, dogs, birds, and *eno* “lie” used with other subjects in Manam, referred to above in Section 4.3.

## 5 CONCLUSION

The basicness and salience of the three primary postures can be viewed as motivating the reliance on one or more of the morphemes with meanings of “sit”, “stand”, and “lie” in a variety of linguistic patterns. The differences between the postures in overall shape, physiology, social function, and so forth can also be viewed as motivating differences in the linguistic behaviors of the corresponding posture morphemes in languages. In the overview presented here, the relevant linguistic phenomena involve both the literal and extended uses of these morphemes. It can be difficult to make the case for extralinguistic motivation of linguistic phenomena, especially when it comes to motivation based on ordinary human experience. Making the case for any experiential motivation for linguistic phenomena is, however, strengthened by the number and diversity of examples and the overview presented here has, I hope, given an indication of just how pervasive these patterns are in languages.

All of the claims about motivation reported here are in need of further study and testing. Some of the linguistic patterns are widespread and have been extensively documented cross‐linguistically, such as the locative and aspectual extensions. Others are in need of further cross‐linguistic substantiation, such as frequency of usage. It seems unlikely that human postures such as, say, standing on tiptoes, standing upside down, or supporting oneself by fingertips would ever function as archetypes for conceptualizing location or duration of objects, but there is still more to be learned about the full range of human positions and their linguistic reflexes, such as squatting as opposed to sitting on the buttocks. The “sit” morpheme would appear to be the most common default posture morpheme in locative and aspectual extensions, also as a source for a “dwell” meaning, but again this idea is in need of further study. In Lakota (Mississippi Valley Siouan, USA), for example, it is the “stand” morpheme, not “sit”, that has been grammaticalized to be the standard continuative auxiliary (Rankin, 2004, pp. 204–205). The gradation in sensorimotor control of standing > sitting > lying and its linguistic reflexes also invite further study, with a view towards finding counterexamples where, say, a “lie” morpheme displays more agentive properties in a language than do the “stand” and “sit” morphemes. The contrast between the dynamic act of moving into an at‐rest position versus the static at‐rest position itself should also be explored further, especially the different extensions that the dynamic versus static senses of the posture morphemes support (cf. Lesuisse & Lemmens, 2018; Newman, 2009).

Finally, the overview presented here can be viewed as an illustration of a number of features that typify research on language and cognition as carried out in the cognitive linguistic approach. The very idea of seeking motivation for linguistic phenomena in broader cognitive realities such as human experience is itself a leading idea within cognitive linguistics. Experiential realities of the human body and how these realities shape linguistic phenomena illustrate further the idea of embodiment, that is, the close relationship between the body and the mind, that has long been a focus of research in this approach. In addition, the range of linguistic evidence underlying the discussions in the preceding sections points to other key concepts and methods in cognitive linguistics. Approaching the posture verbs “sit”, “stand”, and “lie” as prototypes, or conceptual archetypes, connects with ideas about the structure of human cognition that have played a part in cognitive linguistics since its foundation. Exploring the experiential realities associated with the primary postures experimentally, as reported on in Section 3, illustrates the relevance of experimental approaches in cognitive linguistics. The kind of research undertaken by Gibbs et al. (1994) is particularly relevant in this respect, exploring as it does the cognitive realities of postures in a highly targeted way, and provides an experimental design for further experimental work. The reliance on corpora of contemporary language usage, as in Section 4.1, reflects the acceptance of language use as relevant data to language theorizing in cognitive linguistics, and along with that, an interest in corpus‐based methodologies. The discussion of the relatedness of senses, as found in the extensions of postural meanings to other lexical meanings like “sleep” or to grammatical meanings like progressive aspect in Section 4.3, 4.6, illustrates a long‐standing interest in lexical semantics, especially the individuation of word senses and the relatedness of word senses, in the field.

## CONFLICT OF INTEREST

The author has declared no conflicts of interest for this article.

## RELATED WIREs ARTICLE

Semantics and cognition

## Data Availability

Data sharing is not applicable to this article as no new data were created or analyzed in this study.
